# A case of peri-implantitis and osteoradionecrosis arising around dental implants placed before radiation therapy

**DOI:** 10.1186/s40729-016-0039-1

**Published:** 2016-04-05

**Authors:** Yuji Teramoto, Hiroshi Kurita, Takahiro Kamata, Hitoshi Aizawa, Nobuhiko Yoshimura, Humihiro Nishimaki, Kazunobu Takamizawa

**Affiliations:** Department of Dentistry and Oral Surgery, Shinshu University School of Medicine, 3-1-1, Asahi, Matsumoto, 390-8621 Japan

**Keywords:** Dental implant, Radiation therapy, Osteoradionecrosis

## Abstract

A little is known about the effect of radiotherapy on the dental implants that have previously been osseointegrated and charged. Here, we reported a case of osteoradionecrosis which arose around dental implants placed before radiation therapy.

## Background

Osteoradionecrosis (ORN) of the mandible is a severe complication that follows ionizing radiation therapy in patients undergoing treatment for head and neck cancer. The radiation dose, tumor location, dental trauma, premorbid state of dentition, and concomitant chemoradiotherapy are thought to be contributing factors for ORN [1–3]. Most patients with head and neck cancer are aged 50 years or more and include those who have dental prosthetic implants [4]. Dental implant surgery and/or the peri-implant tissue condition might represent a possible etiology for ORN. Many researchers have studied and reported the effects of irradiation on dental implant therapy. Most of them have discussed the effect of previous irradiation on the success or failure of the dental implant rehabilitation [5–7]. In these cases, the dental implant was installed in the irradiated bone. In contrast, little is known about the effects of radiotherapy on dental implants that have previously been osseointegrated and charged [8, 9]. Here, we reported a case of ORN that arose around dental implants placed before radiation therapy.

## Case presentation

**Fig. 1:**
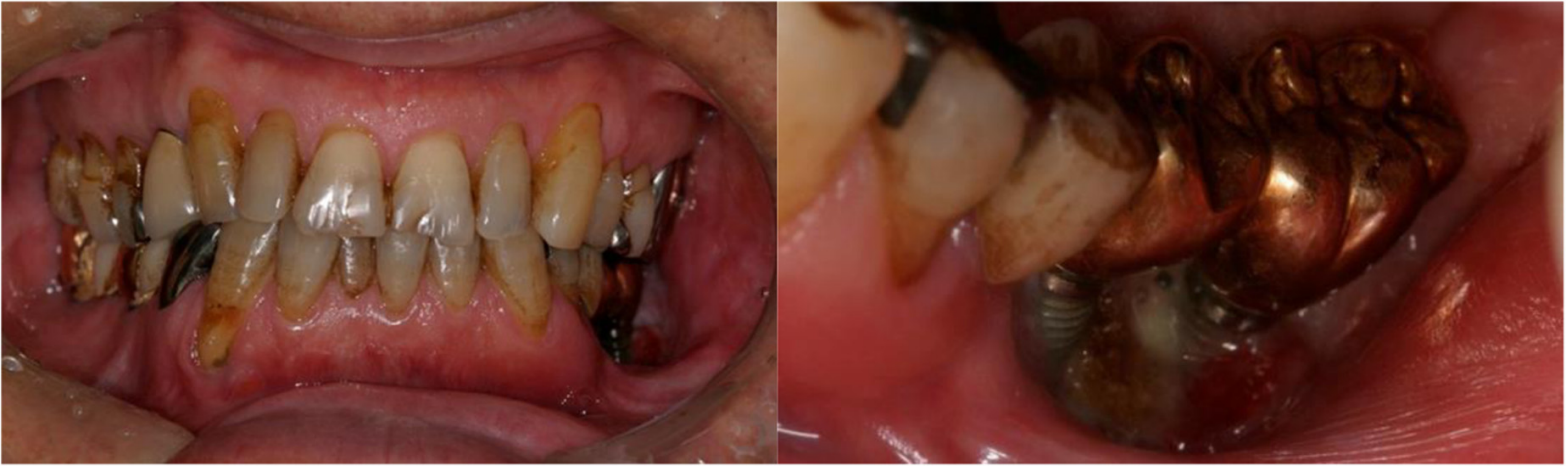
Intraoral photo at the first visit

**Fig. 2:**
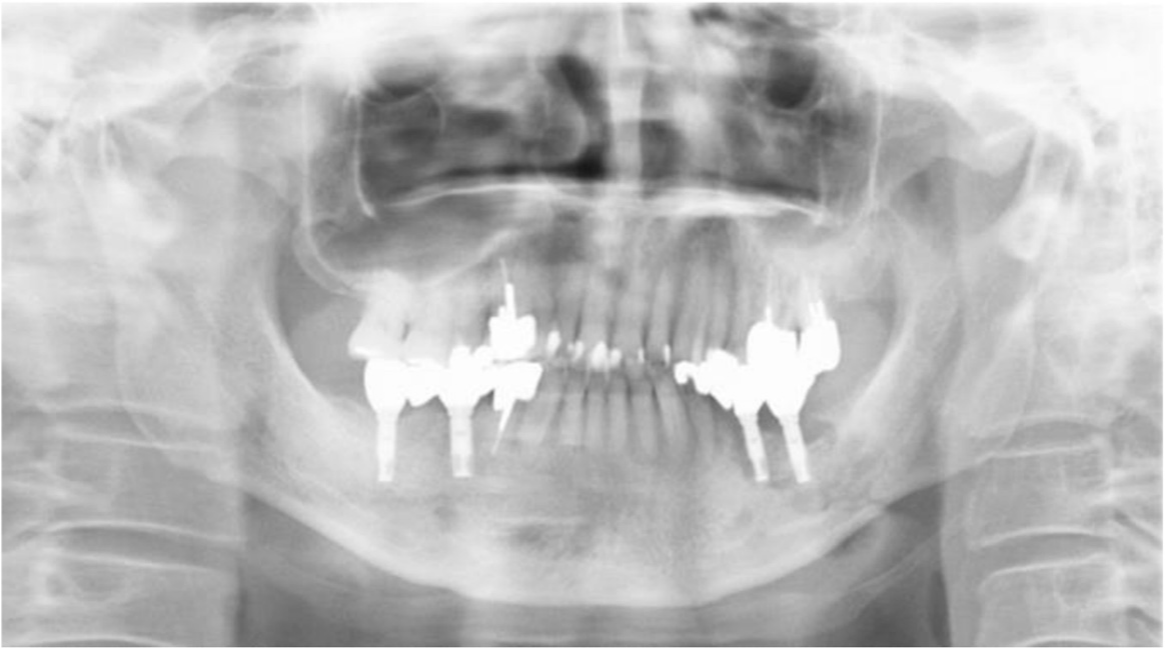
Panoramic X-ray image at the first visit

**Fig. 3:**
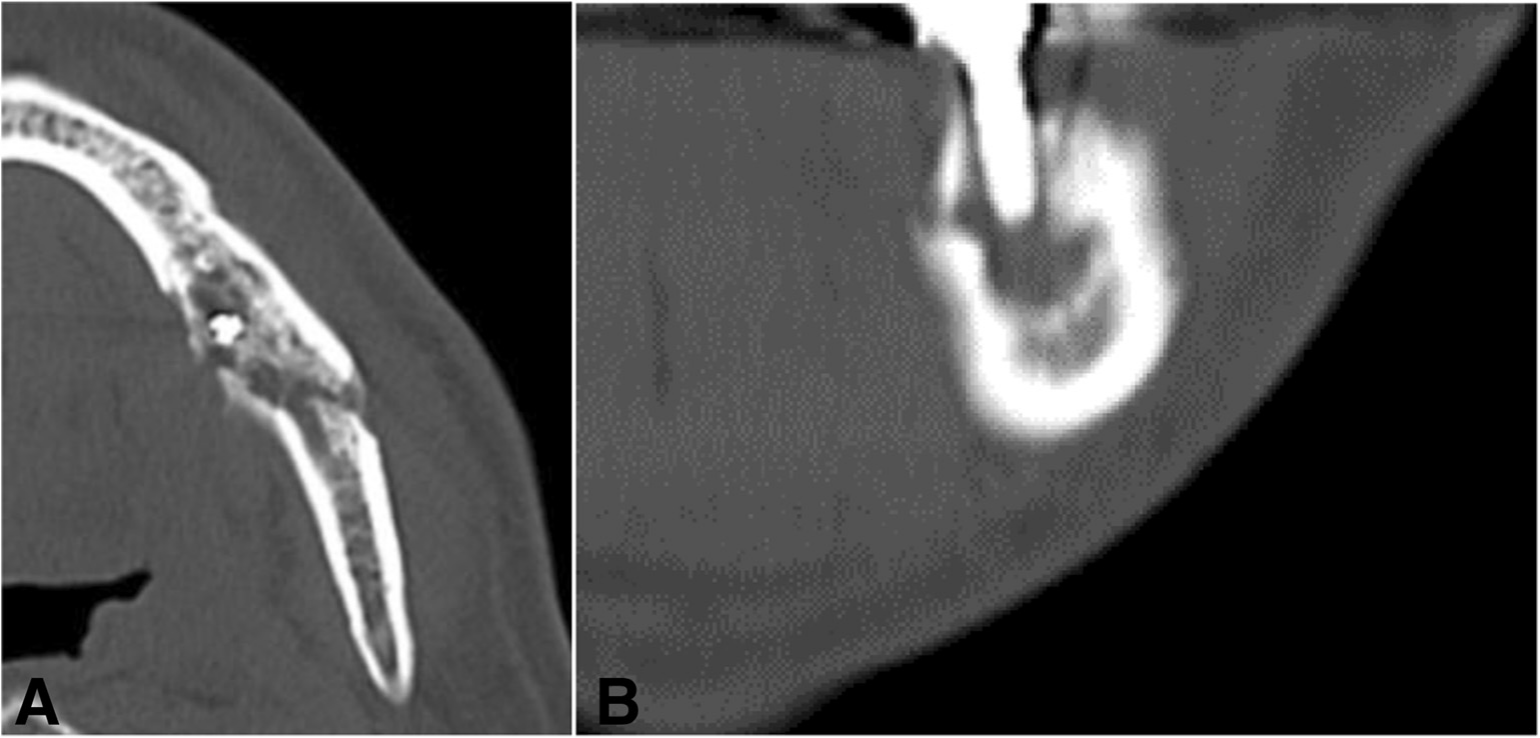
CT images of the left mandible. **a** Axial view at the left first molar. **b** Coronal view at the left first molar

A 66-year-old man was referred to our hospital for further treatment of ORN of the mandible. He had undergone dental implant treatments on both sides of the mandible (#35, #36, #45, and #47) 7 years previously. All of the implants were osseointegrated and charged. The patient had been followed up regularly by his dentist, and the clinical course had remained uneventful. He experienced left oropharyngeal carcinoma and was treated with external radiotherapy of total dose 70 Gy 2 years after the implant treatment. Medical records revealed that his left mandible was included in the radiation field. He began to experience spontaneous pain and gingival swelling around the left mandibular implants 4 years after the oncologic radiotherapy. Under a clinical diagnosis of peri-implantitis, conservative treatment consisting of local irrigation and intermittent use of antibiotics had been carried out for 6 months. However, the symptoms became more serious and bone exposure around the dental implants appeared. He was then referred to our hospital for further treatment. Clinical examination revealed painful left cheek swelling with hypoesthesia of the left lower lip. The mouth opening range was restricted (1.5 fingerbreadth). Intraorally, exposed necrotic alveolar bone surrounding the left mandibular dental implants associated with mucosal inflammation and purulent discharge was observed (Fig. 1). On a panoramic X-ray image, poorly demarcated bone destruction around the left mandibular dental implants (fixtures at #35 and #36) was revealed, and the lesion reached the inferior border of the mandible and caused pathological fracture. No remarkable findings were observed around the right mandibular dental implant (Fig. 2). On CT examination, the mandibular bone was destroyed entirely in the left molar region and a fracture line across the mandible was evident (Fig. 3).

**Fig. 4:**
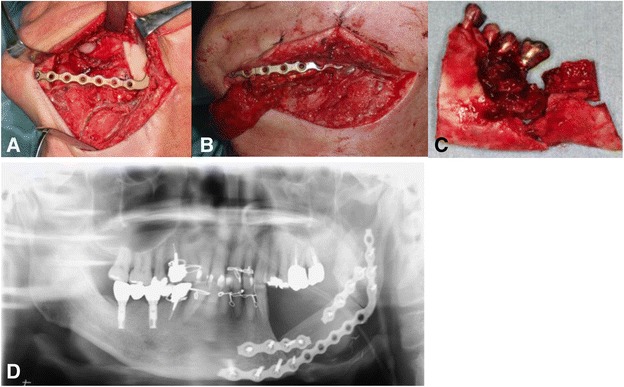
**a** Intraoperative photo. The affected left mandible was segmentally resected. **b** Intraoperative photo. A vascularized fibula bone graft. **c** Resected mandible. **d** Panoramic X-ray image after the surgery

**Fig. 5:**
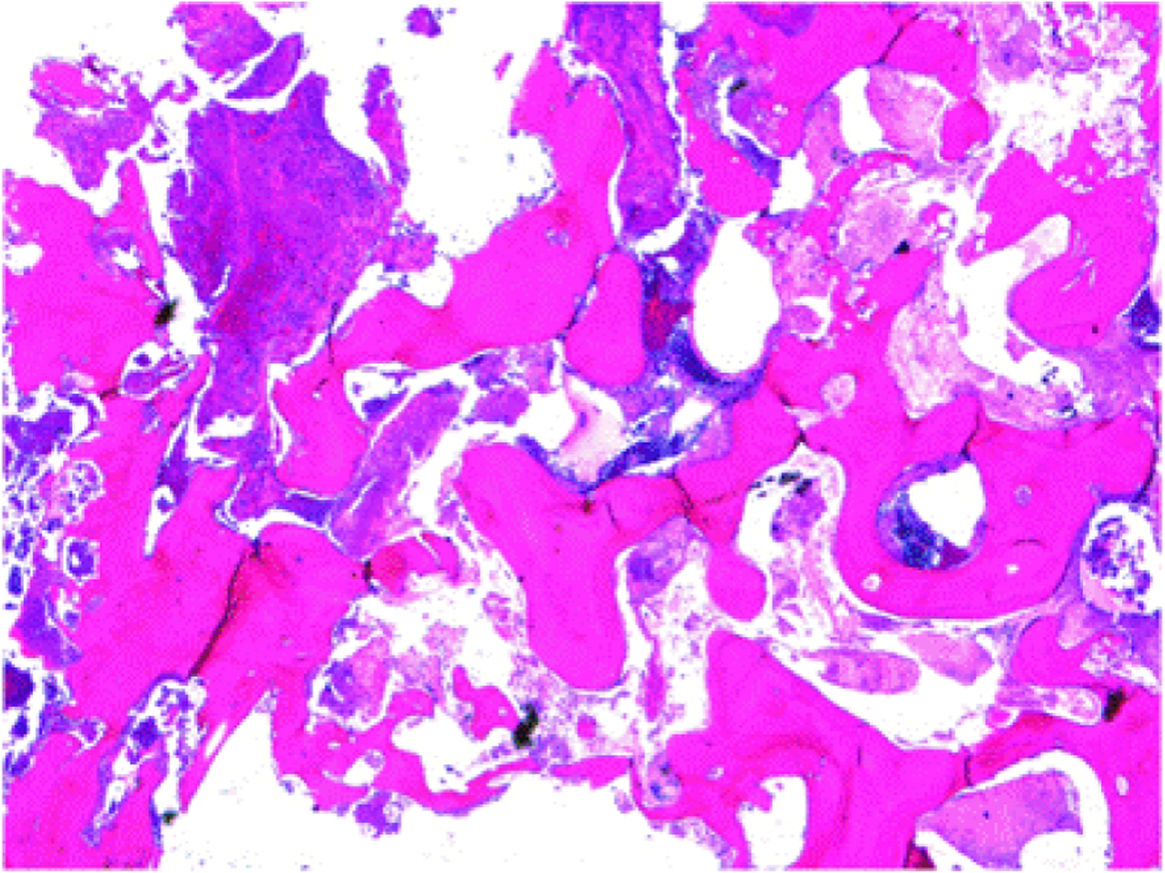
Histopathologic photo of the resected mandible (H-E staining)

**Fig. 6:**
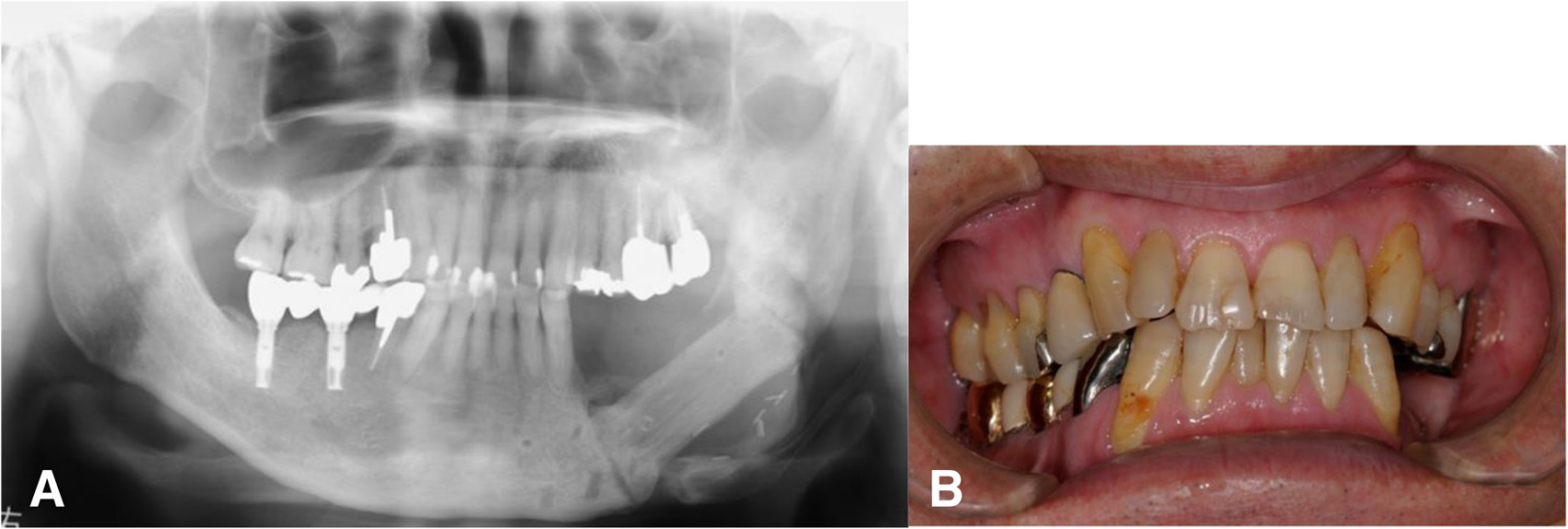
**a** Panoramic X-ray image 1 year after the surgery. **b** Intraoral photo 1 year after the surgery

Initially, at our hospital, 30 sessions of hyperbaric oxygen therapy were carried out, but this resulted in only a slight improvement of symptoms. Thereafter, as the imaging studies proved no further progression of ORN, segmental mandibular resection and simultaneous reconstruction with a fibula free microvascular flap was performed. The affected tissue was determined and harvested based on the radiological changes in addition to the intraoperative macroscopic appearance of the bone. A vascularized fibula bone graft was fixed with a titanium reconstruction plate (Fig. 4). Histopathological assessment of the resected mandible showed loss of osteocytes and osteoblasts and filling of the bony cavities with fungus mass and inflammatory cell infiltration with fibrosis (Fig. 5). A final diagnosis of osteoradionecrosis of the mandible was confirmed. The postsurgical course was uneventful, and long-term follow-up has been successful. There have been signs of neither implantitis nor ORN around the right dental implants (Fig. 6).

### Discussion

In this paper, we reported a case of ORN arising around dental implants placed before radiotherapy. This is the third such case report to be published. Granström et al. reported three cases of ORN developing around dental implants previously placed for skin-penetrating prosthesis [8]. Slama et al. reported a case of mandibular ORN in post-implant radiation [9]. In these cases, the presence of dental implants in the radiation field was thought to play a possible role in the development of ORN. In our case, although the patients had dental implants in both sides of the mandible, ORN occurred only in the left side, which was included in the field of radiotherapy applied for the treatment of oropharyngeal cancer arising in the left side. This fact suggested a possible causal relationship between the presence of dental implants and the development of ORN.

The presence of a dental implant may affect the development of ORN by two mechanisms. Firstly, the presence of the implant may cause a change in the radiation dose distribution around the dental implant [4, 10, 11]. Radiation scatter and electronic disequilibrium from implant materials are thought to cause both soft and hard tissue complications in the oral cavity. Friedrich et al. [10] reported on titanium dental implants in the field of ORN. Ozen et al. [4] examined the dose enhancement from scattered radiation at bone-dental implant interfaces. They reported that there is a 21 % maximum increase in the dose to alveolar mandibular bone in close proximity to the titanium. The increase in dose enhancement fell off rapidly and become insignificant at 2 mm from the interface. They suggested that it is not clear whether a local overdose of the order of 15 to 21 % will cause a significant increase in the incidence of bone necrosis around osteointegrated titanium implants. Beyzadeoglu et al. [11] also reported that irradiation, with different radiation beams and different irradiation angles, did not sufficiently affect the total dose to lead to ORN of the mandible. From the results of these studies, it could be said that the bone-implant interface is exposed to possible dose enhancement from scattering by the dental implants if the dental implants are included in the radiation field.

Secondarily, infection associated with dental implant may become a possible cause of ORN. In radiotherapy including the oral cavity, gingivitis is frequently observed adjacent to fixed metal dental restorations because they cause significant dose enhancement around them [12]. It is easy to speculate that the same occurs around dental implant prostheses (peri-implant mucositis). The presence of mucositis (gingivitis) causes poor oral hygiene and leads to a vicious spiral of poor oral hygiene and increased bacterial infection. Radiation therapy may also interfere with normal wound healing mechanisms. Changes in vasculature, effects on fibroblasts, and varying levels of regulatory growth factors result in the potential for altered wound healing. Radiation also induces alterations of the immune response (immunosuppression). Ionizing radiation directly affects the immune system. These conditions reduce the peri-implant tissue resistance to oral bacteria, thus increasing the risk of peri-implantitis. Slama et al. [9] reported the existence of peri-implantitis prior to the development of ORN. In our case, the peri-implantitis progressed to ORN. It is well known that the defense function of peri-implant tissue is weaker than that surrounding natural teeth. It is likely that the peri-implant mucositis caused by radiotherapy can easily progress to infected peri-implantitis and subsequently more severe infection of the jaw bone.

## Conclusions

Dental implants have become increasingly popular, and a considerable number of people have undergone dental restorations using dental implants. Therefore, there will be an increasing probability of patients with dental implants receiving irradiation around their implants. Further studies are required to analyze whether dental implants located in the radiation field cause adverse effects in the long term.

## Consent

Written informed consent was obtained from the patient for the publication of this report and any accompanying images.
